# Notes of Surgical Operations

**Published:** 1859-10

**Authors:** 


### EDITORIAL.

### NOTES OF SURGICAL OPERATIONS.

Under this head we propose to give from time to time, each month, when our space allows, a brief summary of the most interesting surgical cases coming under our observation during the month, with remarks and references to other similar cases heretofore observed. We are induced to offer these notices to our readers from the belief that the facts thus embodied will be interesting and valuable, and that many of these, for want of such notice, would be lost to science; and further, from the belief that the extent of the field for surgical observation in this city is but imperfectly appreciated. The operations of the lumber region, of the commerce of the upper lakes, of the numerous railroads centering at Chicago, of the manufactures of the city and vicinity,—involve a number of accidents as great, perhaps, as are to be met with in any other city of America or Europe, New York alone excepted; while patients with chronic surgical diseases resort hither, to some extent, for advice from five large States and two Territories, embracing a population of many millions.

It is our wish to make this field of observation available for purposes of instruction, for the improvement of the profession, and for the advancement of science to as great a degree as possible. This can only be done by a brief record of the cases as they present themselves, reserving their use in a more permanent form to another occasion.

1. *Foreign Body in the Urinary Bladder.*

We have recently removed a short pencil of plumbago and wood from the bladder of a man somewhat advanced in years, by the lateral operation of lithotomy. He is out of danger. This is our seventeenth operation without accident.

2. *Extirpation of the Globe of the Eye for Cancer.*

This operation was lately performed on a young child, four years old, for cancer of about one year's growth. The capsule of the globe was unaffected, and was left, which renders the operation much less serious than when it is necessary to remove more of the contents of the orbit. This child suffered no severe symptoms after the operation; her general health and the use of the sound eye were, indeed, improved. Many ophthalmic surgeons, in view of the rapid return of the disease, condemn the operation. Walton, in his recent valuable work on the surgery of the eye, leans to this view. However unfavorable the result in this respect may be, it can scarcely be more so than the natural course of the disease. We had recently occasion to examine with Dr. Holmes a case in which both eyes were in the early stage of cancer. It was about four months since, and, at the present time, the disease is near its usual fatal termination.

3. *Trephining for Epilepsy and Insanity.*

This operation was performed on a man, thirty years of age, affected with epilepsy and some mental derangement for about four months. The point chosen was the left side of the occiput below the protuberance, the seat of a tumor which had existed for many years, and caused an absorption of the bone and a roughening of its surface. The two tables at the point of the operation were found consolidated and of an ivory hardness, and the skull of but half its natural thickness. The dura mater was vascular and adherent to the pia mater. The patient, who had been under the advice of Dr. Chambers, of Charleston, Coles Co., Ill., returned home three weeks after the operation, very quiet, and much improved in his health. If kept from the exciting causes of insanity, there can be little doubt of his entire recovery.

We have notes of five other cases in which this operation was performed for epilepsy, accompanied by more or less alteration of the mental faculties.

The first of these was that of a young man, John Ladrigan, cut upon the head at two points by an axe in the forests of Wisconsin. One wound extended from the median line, at the junction of the coronal and sagittal sutures, to the left side three inches. The edge of the axe penetrated the substance of the brain deeply, portions of which escaped. The other cut, also on the left side, followed the line of junction of the parietal and occipital bones, and was as deep and as long as the other. At each a large piece of bone was partially separated, one edge passing upon the brain, and the other rising above the surface of the cranium.

This man stated that he had been left as in a hopeless condition by the physicians who were called to see him, but recovered with the wounds in the condition we have mentioned, the right superior and inferior members being affected with paralysis and contraction. He was also subject to very frequent and severe paroxysms of epilepsy, which occurred sometimes daily.

This man was operated on before the class of Rush Medical College.

The whole of the displaced fragment of bone at the seat of the anterior wound was removed by three applications of the crown of the trephine. He recovered perfectly; had but one slight epileptic paroxysm afterwards, and gradually recovered considerable use of the members. No operation was performed on the posterior wound.

The second case was that of a young man from Kendall Co., Illinois. He received a blow above the ear, which fractured and depressed the skull without dividing the scalp. No operation was performed at the time, and he recovered, as was supposed. Six months afterwards he was seized with an epileptic fit. Three months afterwards, another occurred, and then one every month. One year after the injury, we removed the depressed portion with the trephine. He recovered, and the epileptic paroxysms recurred at lengthening intervals, until, at the last notice, six months had elapsed without a return.

The third case was that of a young man, kicked by a horse above the ear, fracture and depression being produced. Concussion and insensibility were the immediate results; after recovering from which, he remained insane, or nearly so. About four weeks after the injury, he was admitted into the wards of the so-called Mercy Hospital, where I used the trephine. Removing the depressed portions of bone gave immediate relief.

The fourth case was a man of fifty years, who, eleven years previously, had a fracture with depression in the temporal region. This gave rise for several years to no perceptible inconvenience. At length he began to be affected with giddiness and loss of consciousness, which, by degrees, became more frequent, until the severer forms of epilepsy were developed. The mind was also affected by dementia to a degree which disqualified him for business. Removing the depressed pieces of bone gave great relief, and one year after the operation he was steadily improving.

The fifth case was not so favorable. A lad, about sixteen years old, came for advice in regard to severe epileptic attacks which had continued for several years. His friends stated that when an infant he had received a blow on the left superior part of the os frontis. On examining this part of the head, a depression was perceived, which, however, was soft and easily compressible. Although no bony surface could be felt at this point, yet the statement of friends of the patient that the blow had produced a depression, induced me to apply the trephine upon its margin. It was found, however, that the bone at that point was entirely absorbed; that surrounding it was in a condition of hypertrophy, very spongy and vascular, and three times its natural thickness. The soft parts presented this appearance in the point where the bone was deficient of venous erectile tissue.

In this case no benefit appeared to result from the operation.

From the four preceding, the inference is, I think, deducible, that leaving depressed pieces of cranium when there is neither wound of the scalp nor symptoms of compression of the brain, as is advised by most surgical writers, is attended by dangers not usually suspected, and that it is better, in all such cases, to raise up the depressed portions at once.

4. *Reduction of Dislocations of the Hip by Manipulation.*

A case of dislocation of the hip into the ischiatic notch came under our care, Friday, Sept. 17, which was readily reduced by following exactly the direction of Reid. It is the first time we have succeeded by this plan. The only other case of a similar kind in which we have attempted it was a dislocation on the dorsum of the ilium of six weeks' standing. Bearing in mind the fracture of the cervix femoris, said to have been produced by too forcible attempts of this kind by a surgeon of the N. Y. Hospital, we took care not to use too great force.

We seize the occasion, however, to mention some cases of dislocation into the thyroid foramen, reduced without pulleys or other mechanical power. The first of these occurred several years since, at Michigan City, Indiana. Attempts had already been made by a surgeon, who tried the pulleys and Jarvis' adjuster without success. We applied the pulleys, but the force deemed as much as could safely be used was insufficient. We then wound a stick of wood with a piece of quilted stuff, to about six inches in diameter, and having it pressed by an assistant against the perineum and firmly held between the thighs, we seized the ankles, and, the knees being straight, carried the two members towards and in a manner tending to cross each other, using the piece of wood as a fulcrum and the members as levers. The dislocation was readily reduced.

Since then two other cases of the same accident have been treated in the same manner with success. In one the patient was laboring at the time of reduction under the effect of shock from falling from a height. The bed-post was surrounded by a pillow, the patient drawn down so as to fetch this against the perineum, and the same manipulation used as before. The force required in this case was not great. In all these cases, except the first, the patients were placed fully under the effects of chloroform.

Judging from these, and a case in which we recently reduced a dislocation of the shoulder without the aid of assistants or mechanical means, it seems doubtful whether the force of the surgeon skilfully applied is not, with the aid of chloroform, sufficient to reduce any dislocation whose reduction ought to be attempted.

